# *Strigea robusta* causes polydactyly and severe forms of Rostand’s anomaly P in water frogs

**DOI:** 10.1186/s13071-020-04256-2

**Published:** 2020-07-29

**Authors:** Anton O. Svinin, Ivan V. Bashinskiy, Spartak N. Litvinchuk, Oleg A. Ermakov, Alexander Yu. Ivanov, Leonid A. Neymark, Aleksander A. Vedernikov, Vitalij V. Osipov, Galina P. Drobot, Alain Dubois

**Affiliations:** 1 Mari State University, 424000 Yoshkar-Ola, Russia; 2 A.N. Severtsov Institute of Ecology and Evolution, Russian Academy of Sciences, 119071 Moscow, Russia; 3 Institute of Cytology, Russian Academy of Sciences, 194064 St. Petersburg, Russia; 4 Dagestan State University, 3367000 Makhachkala, Russia; 5 Penza State University, 440026 Penza, Russia; 6 Privolzhskaya Lesosteppe State Nature Reserve, 440031 Penza, Russia; 7 Russian Federal Research Institute of Fisheries and Oceanography, 410002 Saratov, Russia; 8 Muséum National d’Histoire Naturelle, Institut Systématique, Evolution, Biodiversité, 75005 Paris, France

**Keywords:** Anomaly P, *Pelophylax*, Trematodes, *Strigea robusta*

## Abstract

**Background:**

Cases of polydactyly in natural populations of amphibians have attracted great interest from biologists. At the end of the 1940s, the French biologist Jean Rostand discovered a polymorphic syndrome in some water frog (Anura: *Pelophylax*) populations that included polydactyly and some severe morphological anomalies (he called it ‘anomaly P’). The cause of this anomaly remains unknown for 70 years. In a previous study, we obtained anomaly P in the laboratory in tadpoles of water frogs that developed together with molluscs *Planorbarius corneus* (Mollusca: Gastropoda) collected in the field. We thus proposed the ‘trematode hypothesis’, according to which the infectious agent responsible for anomaly P is a trematode species.

**Methods:**

Metacercariae from tadpoles with anomaly P were identified using ITS2 gene sequencing as *Strigea robusta* (Trematoda: Strigeidae). To verify teratogenic features of the species, cercariae of *S. robusta* were tested for the possibility to cause anomalies. Identification of cercariae species was made using morphological and molecular methods (sequencing of ITS2 and *28S* rRNA). The tadpoles were exposed to parasites at four doses of cercariae (control, low, medium and high) and divided into two groups: “early” (at 25–27 Gosner stages) and “late” (at 29–34 Gosner stages) exposure.

**Results:**

A total of 58 (72.5%) tadpoles survived until metamorphosis under the dose-dependent experiment with the trematode *S. robusta*. Differences in the survival rates were observed between the exposed and unexposed tadpoles both in the group of “early” tadpoles and “late” tadpoles. The exposure of tadpoles to the cercariae of *S. robusta* induced anomaly P in 82% of surviving tadpoles. The severe forms developed only in “early” stages under all doses of cercariae exposure. Polydactyly predominantly developed in the “late” stages; under a light exposure dose, polydactyly also developed in “early” tadpoles. Laboratory-hatched tadpoles reared together with infected snails had different rates of survival and complexity of deformations associated with the period of coexistence.

**Conclusions:**

The experiments with direct cercariae exposure provide compelling evidence that *S. robusta* leads to anomaly P in tadpoles of water frogs. The manifestation of anomaly P turned out to be dependent on the stage of development, cercariae dose, and the location of the cysts.
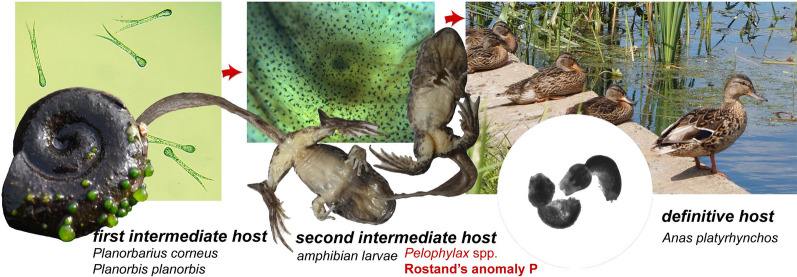

## Background

In amphibians, polydactyly (together with ectromely, ectrodactyly and polymely) is one of the most frequent skeletal anomalies (figure 36b in [1]). It consists of various numbers of extra digits or parts thereof, and can occur in hindlimbs, forelimbs, or both, and be symmetrical or asymmetrical [2]. Polydactyly can manifest itself in different types of morphological anomalies: schizodactyly (duplication of a digit’s part); synpolydactyly (fusion of proximal parts of a duplicated digit); hyperdactyly (duplication of a full digit); and polyphalangy (additional phalanges) [1, 2]. About 40 records of polydactyly are known in water frogs of the genus *Pelophylax* across Europe [3, 4]. However, the etiology of this anomaly remains unknown in many cases [1].

In 1947, the famous French writer and biologist Jean Rostand started to study polydactyly in anurans. From 1947 to 1951, he demonstrated the existence of several types of polydactyly resulting from recessive and dominant mutations in common toad (*Bufo bufo*) populations of the Paris region [5]. In 1949, he found mass symmetrical polydactyly in hind limbs (14.5% of adults) in a water frog population (then known as “*Rana esculenta*”) in Trévignon, western France [6]. In 1952, while studying tadpoles from the same locality, he found new morphological anomalies, including some very severe cases: strongly modified hind limbs with symmetrical flexions (taumely); polydactyly; edemas; brachymely; outgrowths; small additional limbs and spikes; and polydactyly in forelimbs [7, 8]. He noted a gradient in these abnormal structures and anomalies, starting with polydactyly as its mildest expression, and proposed that they clearly belonged to the same syndrome, which was termed ‘anomaly P’ [9]. Unlike in *Bufo*, this anomaly was demonstrated to not be inheritable and was then supposed to be determined by an environmental agent acting on the very early stages of tadpole development (after hatching). For 20 years, Rostand tried in vain to find the cause of the anomaly. He successfully obtained anomaly P in experiments involving fishes (tadpoles reared with fish and content of fish intestine) and thus supposed that this syndrome was caused by a teratogenic virus transmitted by fish or any components of fish diet [8–10]; however, the evidence in favor of this hypothesis was scant. In 1976, Dubois did not succeed to repeat these last results of Rostand [10].

In 2016 and 2017, mass polydactyly and severe cases of anomaly P (17.6%) were found in populations of the marsh frog (*Pelophylax ridibundus*) in the European part of Russia [11]. Severe cases of anomaly P were revealed again for the first time in 40 years and were registered in 6.2% of individuals [4, 11]. Despite chemical pollution of water (above-threshold concentrations of heavy metals and biogenic elements), authors suggested a biological nature of the infectious agent [11] of the anomaly following Rostand [9]. Additionally, three new populations of *P. lessonae* and *P. ridibundus* with individuals showing anomaly P, were found in Russia [4]. In one of these populations, discovered in 1998, anomaly P has disappeared, as has happened with all “ponds with monsters” studied in France in 1952–1971 [9]. Finally, anomaly P was obtained in laboratory experiments [12] where water frog tadpoles were raised in tanks with the mollusc *Planorbarius corneus* (Mollusca: Gastropoda) collected in the field. In tadpoles with the anomaly, cysts with metacercariae of trematodes were detected. Based on this, the ‘trematode hypothesis’ was proposed according to which an infectious agent responsible for anomaly P is a trematode species [11].

Recent studies have shown that, in several amphibian species, the anomalies can be caused by some species of trematodes [13–25]. The most investigated cases concerned the trematode *Ribeiroia ondatrae* in many amphibian populations across the USA and Canada [13–15]. This species induces the development of additional complete or partial limbs, as well as other anomalies, reducing mobility of amphibians and making them easier prey for birds or mammals as definitive hosts [26]. Large-scale research showed a strong association of these deformities with *Ribeiroia* infection over a wide area across the USA [27, 28]. The effect of cercariae exposure on different stages of larval development leads to different manifestations of anomalies [29]. The sensitivity to *Ribeiroia* infection can be different among various amphibians. Species with more rapid larval development, which are smaller at metamorphosis (‘fast-species’), are more susceptible to the infection [30].

Although there are many records of abnormal amphibians in Eurasia [1], only some cases of trematode-dependent anomalies were strongly supported by laboratory experiments [20–25]. Here, we describe new variants of morphological anomalies caused by the trematode *Strigea robusta* in Eurasian amphibians. This discovery might explain the high number of polydactyly records in western Palaearctic water frogs observed in different localities in Europe.

## Methods

### Field sites and samples

We collected individuals of the marsh frog (*P. ridibundus*) in 2016–2019 in Ostrovtsovskaya Lesosteppe (52.815426°N, 44.458827°E, part of the Privolzhskaya Lesosteppe Nature Reserve, Penza Province, Russia). In total, 432 individuals of different age groups were collected: 320 tadpoles, metamorphs, and juveniles; 60 subadults; and 52 adults. Among them, in 2019, we collected 11 tadpoles and 37 juveniles. Ten studied water bodies were described previously [11]. Species identification of water frogs was achieved by multiplex PCR analysis described previously by Ermakov et al. [31].

A helminthological study was performed on four ducks (*Anas platyrhynchos*) from adjacent territories with Ostrovtsovskaya Lesosteppe in August, 2019; one was infected with 40 adult worms of *S. robusta*.

### Experimental tadpoles

Experimental tadpoles were obtained by a laboratory crossing of species of the genus *Pelophylax*. In May and June 2019, the water frogs were collected from three localities in Mari El Republic, Russia: Krasnooktyabrskiy settlement (56.681188°N, 47.679397°E), Ilynka settlement (56.801795°N, 47.908759°E), and Kuguvan settlement (56.788572°N, 47.783403°E). Anomaly P had never been observed in these localities. We performed 3 crosses of 6 individuals: 2 crosses among pairs of *P. lessonae* and 1 cross of a male *P. esculentus* and female *P. lessonae*. Species identification was carried out using the multiplex PCR method [31].

### Experimental cercariae

Metacercariae from tadpoles with anomaly P, which were reared in the laboratory with the mollusc *P. corneus* [12], were identified by molecular analysis as *S. robusta* (see Results). Dose-dependent experiments with cercariae of this trematode species were carried out.

Freshwater snails were collected from ponds with a high frequency of anomaly P in water frogs (Ostrovtsovskaya Lesosteppe) during 2018–2019. Our previous laboratory experiments [12] showed that anomaly P can be obtained in tadpoles reared together with the mollusc *P. corneus*. Another mollusc, *Planorbis planorbis*, was used for cercariae screening because it was registered as main first intermediate host for *S. robusta* [32]. A total of 1316 *P. corneus* and 851 *P. planorbis* were used for cercariae screening. The molluscs were placed individually in small containers (50 ml) with aged tap water. The emergence of cercariae was stimulated by heating the containers with a lamp for 1‒2 h; the distance between the lamp and container was approximately 15–20 cm. The cercariae were studied using light microscopy. For their vital staining, neutral red and Nile blue stains were used. The cercariae from each shedding snail were examined using a light microscope (400×). The primary morphological identification of *S. robusta* cercariae was based on Faltýnková et al. [33].

### Molecular analysis of trematodes

We carried out molecular analyses for identification of different life-cycle stages of *S. robusta*. We used two metacercariae (E3A and E6A) from tadpoles with anomaly P [12], cercariae from *P. corneus* used in the dose-dependent experiments, and one adult worm from the intestine of a field-collected mallard. Molecular analysis was performed for cercariae from a snail (*P. corneus*) used in the dose-dependent (see ‘Experimental design’), 3-day and 20-day experiments (for the 30-day experiment we used cercariae identified based on morphological traits from another snail).

DNA was extracted from trematodes using the standard salt-extraction method [34]. The *28S* rRNA and ITS2 (internal transcribed spacer 2) markers were used in the identification of trematode species [35, 36]. The following primers were used for PCR amplification: 3S (5’-GGT ACC GGT GGA TCA CGT GGC TAG TG-3’) and ITS 2.2 (5’-CCT GGT TAG TTT CTT TTC CTC CGC-3’) for ITS2, and dig12 (5’-AAG CAT ATC ACT AAG CGG-3’) and 1500R (5’-GCT ATC CTG AGG GAA ACT TCG-3’) for *28S* rDNA [35, 36]. The PCR reaction mixture (25 μl) contained 50–100 ng of DNA, 0.5 μM of each primer, 0.2 mM dNTPs, 1.5 mM MgCl_2_, 2.5 μl 10× PCR buffer (10 mM Tris-HCl, pH 8.3, 50 mM KCl), and two units of *Taq* polymerase (Thermo Fisher Scientific, Oyster Point, CA, USA). The thermocycling profile for *28*S rRNA was: 30 s denaturation at 94 °C; 40 cycles of 30 s at 94 °C, 30 s at 53 °C, 2 min at 72 °C; and a 5 min extension at 72 °C. The initial cycle for ITS2 was: 32 cycles of 30 s at 94 °C, 30 s at 53 °C, and 45 s at 72 °C. The PCR fragments were prepared for sequencing by elution with a high-salt solution from a 6% polyacrylamide gel. Sequencing was performed on an ABI 3500 automated sequencer (Applied Biosystems, Foster City, CA, USA) using the BigDye®Terminator 3.1 kit (Applied Biosystems) and the same primers used for amplification. The obtained sequences were aligned and edited manually in Chromas v. 2.5.1 (Technelysium, Tewantin, Australia).

The sequences obtained have been deposited in GenBank (ITS2: MT075803, MT075804, MK295777, MK295776; *28S* rRNA: MT075841, MT075842, MK585230, MK585229). Screening of the primary sequences was performed using the BLAST algorithm available through the National Center for Biotechnology Information [37].

### Experimental design

Cercariae of the trematode *S. robusta* were tested for a possibility to cause anomalies. For this, each tadpole was placed in a separate specimen cup containing 50 ml of aged tap water. The tadpoles were then exposed to parasites at 4 doses of cercariae over a 10-day period, following Johnson et al. [13]: 0 (control), 16 (low), 32 (medium) and 48 (high). For the experiment we used ‘fresh’ cercariae after 1–2 h of emergence. We used cercariae from one snail infected with one species of trematode identified by molecular analysis. The number of cercariae was counted using a dissecting microscope, and the predetermined numbers of cercariae were released in each cup. The tadpoles were then kept in the cups for a minimum of 6 h.

The developmental stages of tadpoles were determined according to Gosner’s [38] table of normal anuran development. The tadpoles were divided into two groups: “early” (at 25–27 Gosner stages) and “late” (at 29–34 Gosner stages) exposure according to Holland et al. [16] and Johnson et al. [29] with modifications. After exposure, the tadpoles were transferred into 60 l aquariums. Ten tadpoles of each group (i.e. 0-early, 0-late, 16-early, 16-late, 32-early, 32-late, 48-early and 48-late) were tested. Thus, a total of 60 tadpoles received doses of *S. robusta* cercariae. The water in the aquariums was changed twice a week. Tadpoles were fed fish food (TetraMin Inc., Melle, Germany) *ad libitum*.

In an additional experiment, tadpoles were kept together with *P. corneus* snails infected with *S. robusta* in 60 l aquariums for three different time periods: 3 days, 20 days, and 32 days. The initial sample sizes in each aquarium were 10 tadpoles and one snail. The water in these aquariums was not changed during the period of coexistence.

We described anomalies in tadpoles using a dissecting microscope following the terminology of Henle et al. [2] and specific terminology for anomaly P [8, 10]. We counted metacercariae in tadpoles after the experiment under a dissecting microscope.

### Statistical analysis

Rates of survival and anomalies were analyzed using logistic regression with Firth’s correction [29]: normal or abnormal tadpoles (alternatively, alive or dead) were the response variables; cercariae dose and developmental stage of the tadpole were predictors. The significance of difference between frequency of parasite occurrence in snails was tested by Yates corrected *χ*^2^ test [39]. Because tadpoles from the same treatment were grouped together in aquaria after infection, our samples were not strongly independent. This problem was partly solved by statistical analysis: we used a generalized linear mixed model with a random intercept term for the container (individual tadpoles were used as nested observations). All calculations were performed using the R software and RStudio (v. 1.2.5042).

## Results

### Molecular identification of trematode species

The morphological study of trematode cysts in tadpoles with anomaly P revealed the presence of only one trematode species. Molecular analysis of these cysts allowed us to identify the specific status of this trematode. The sequence of the ITS2 gene of *S. robusta* (GenBank: MF537205 [40]) was identical to our sequences (Table 1). Sequences of the *28S* rRNA gene of *S. robusta* were not represented on GenBank. Our sequences of the *28S* rRNA were 98% similar to *Apharyngostrigea cornu* (GenBank: MF398344). In addition, we sequenced an adult worm of *S. robusta*, determined by morphological traits, and the sequence was identical with metacercariae sequences (Table 1). Thus, according to molecular data, cysts of *S. robusta* were found in tadpoles with anomaly P, and, therefore, this trematode species is the first possible candidate for the role of “infectious agent” of anomaly P. Additionally, cercariae from *P. corneus* collected in ponds with anomaly P were sequenced (*28S* rRNA and ITS2). According to these data, they were identical to the adult worm and metacercariae and also belong to *S. robusta*.

**Table 1:** Sequences deposited in GenBank and similarities of sequences according to BLAST analysis

Stages of *S. robusta* life-cycle	Host	ITS2 GenBank ID	% similarity	*28S* rRNA GenBank ID	% similarity
Cercariae (used in dose-dependent experiments)	*Planorbarius corneus*	MT075803	MF537205 476/476 (100%) *Parastrigea robusta*	MT075841	MF398344 1225/1251 (97.9%) *Apharyngostrigea cornu*
Metacercariae	*Pelophylax esculentus*	MK295777 MK295776	MF537205 469/469 (100%) *Parastrigea robusta*	MK585230 MK585229	MF398344 1227/1253 (97.9%) *Apharyngostrigea cornu*
Adult worms	*Anas platyrhynchos*	MT075804	MF537205 476/476 (100%) *Parastrigea robusta*	MT075842	MF398344 1225/1251 (97.9%) *Apharyngostrigea cornu*

### Survival of tadpoles

**Fig. 1:**
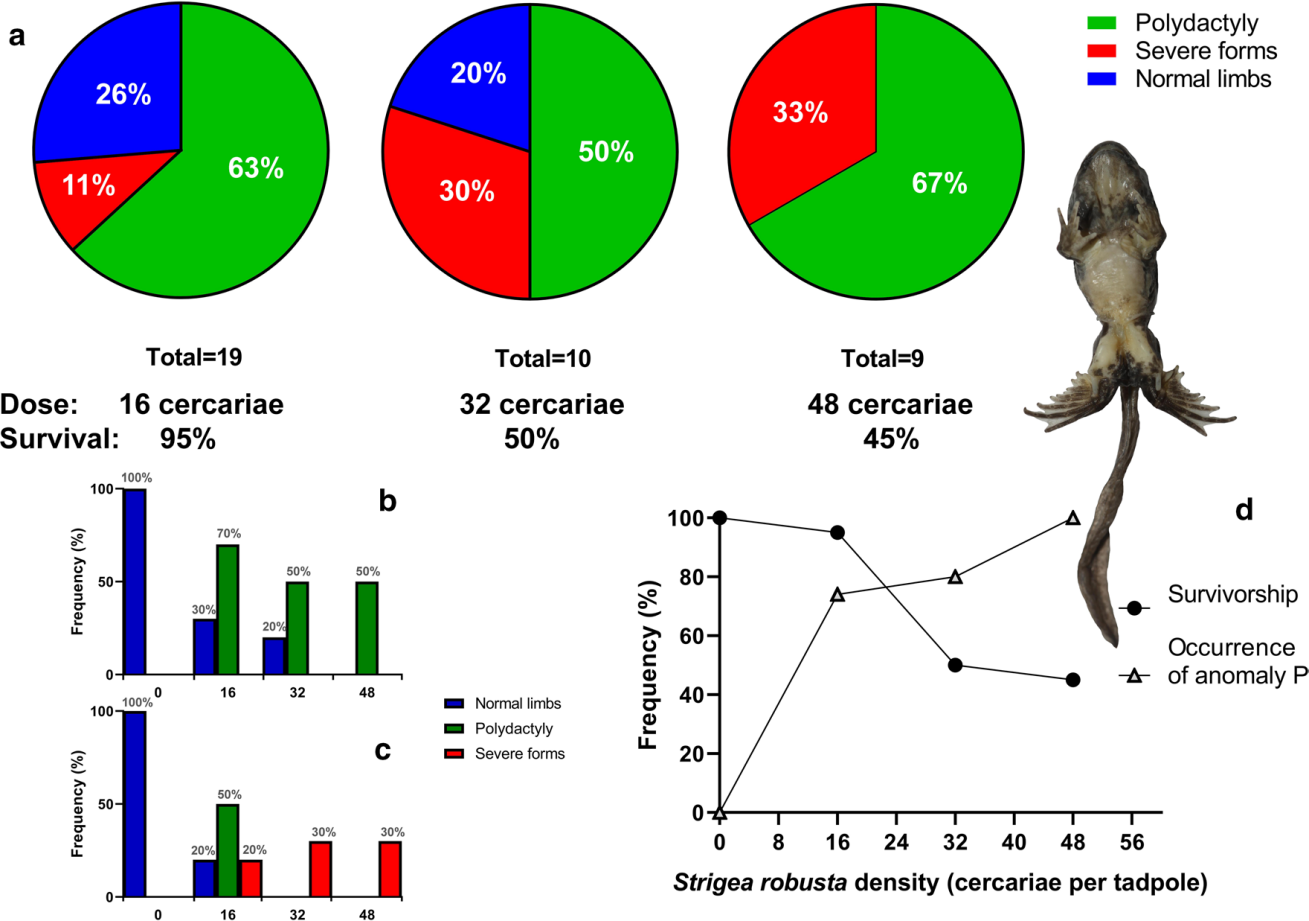
Survivorship, frequency and complexity of the anomaly P in *Pelophylax lessonae* tadpoles infected with *Strigea robusta*. **a** Frequency of mild and severe forms of anomaly P in tadpoles under a given treatment (infection with 0, 16, 32 and 48 cercariae). Initial sample sizes in each treatment were 20 tadpoles. **b**, **c** Complexity of anomalies in tadpoles on “late” (**b**) and “early” (**c**) stages of limb development. Initial sample sizes in each group were 10 tadpoles. **d** Correlation between survivorship and frequency of anomalies in tadpoles

For strong evidence of a teratogenic effect of the trematode *S. robusta*, it was necessary to conduct direct cercariae dose-dependent experiments. A total of 58 (72.5%) tadpoles survived until metamorphosis under the dose-dependent experiment with *S. robusta*. The exposure of tadpoles to cercariae induced anomaly P in 82% of tadpoles that survived until metamorphosis (*n* = 38; see Table 2, Fig. 1). In control groups, all tadpoles (*n* = 20) survived and had no anomalies.

**Table 2:** Composition of anomalies from the laboratory experiment and field collection

Anomaly type	Control (0)		Light (16)		Intermediate (32)		Heavy (48)		Total for treatments 16, 32, 48	Field sites (*n* = 384)
	Early	Late	Early	Late	Early	Late	Early	Late		
Anophthalmy	**–**	**–**	**–**	**–**	**–**	**–**	**–**	**–**	**–**	0.003
Mandibular hypoplasy	**–**	**–**	**–**	**–**	**–**	**–**	**–**	**–**	**–**	0.003
Non-developed operculum	**–**	**–**	**–**	**–**	**–**	**–**	0.10	**–**	0.03^a^	0.008
Brachydactyly	**–**	**–**	**–**	**–**	**–**	**–**	**–**	**–**		0.005
Stiff hindlimbs	**–**	**–**	**–**	**–**	**–**	**–**	**–**	**–**	**–**	0.003
Polydactyly on hindlimbs	**–**	**–**	0.50	0.70	**–**	0.50	**–**	0.60	0.61^a^	0.200
Severe cases of the anomaly P	**–**	**–**	0.20	**–**	0.30	**–**	0.30	**–**	0.21^a^	0.047
Normal tadpoles	1.00	1.00	0.20	0.30	**–**	0.20	**–**	**–**	0.18^a^	0.745
Survival of tadpoles	1.00	1.00	0.90	1.00	0.30	0.70	0.30	0.60	0.63	**–**

^a^Values calculated from a total of 38 surviving tadpoles; non-developed operculum found in a tadpole with heavy variant of the anomaly P

When tadpoles were exposed to a dose of 16 cercariae, almost all individuals survived (19 individuals), but the survival reduced in experiments with larger exposures (32 and 48) and amounted to about 50% (Fig. 1). Statistically significant differences in the survival rates were observed both in the group of “early” tadpoles (logistic regression with Firth’s correction: *χ*^2^ = 18.14, *df* = 1, Wald = 10.3, *P* = 0.001) and the “late” tadpoles (*χ*^2^ = 9.06, *df* = 1, Wald = 5.12, *P* = 0.024).

Tadpoles reared together with infected snails had different rates of survival associated with the period of coexistence. A 3-day period led to 100% survival. Sixty percent of the tadpoles underwent metamorphosis after 20 days of coexistence. Hyperinvasion of *S. robusta*, observed during 20–30 days of coexistence, led to almost total mortality: only one tadpole (10%) survived.

### Manifestation of anomaly P

**Fig. 2:**
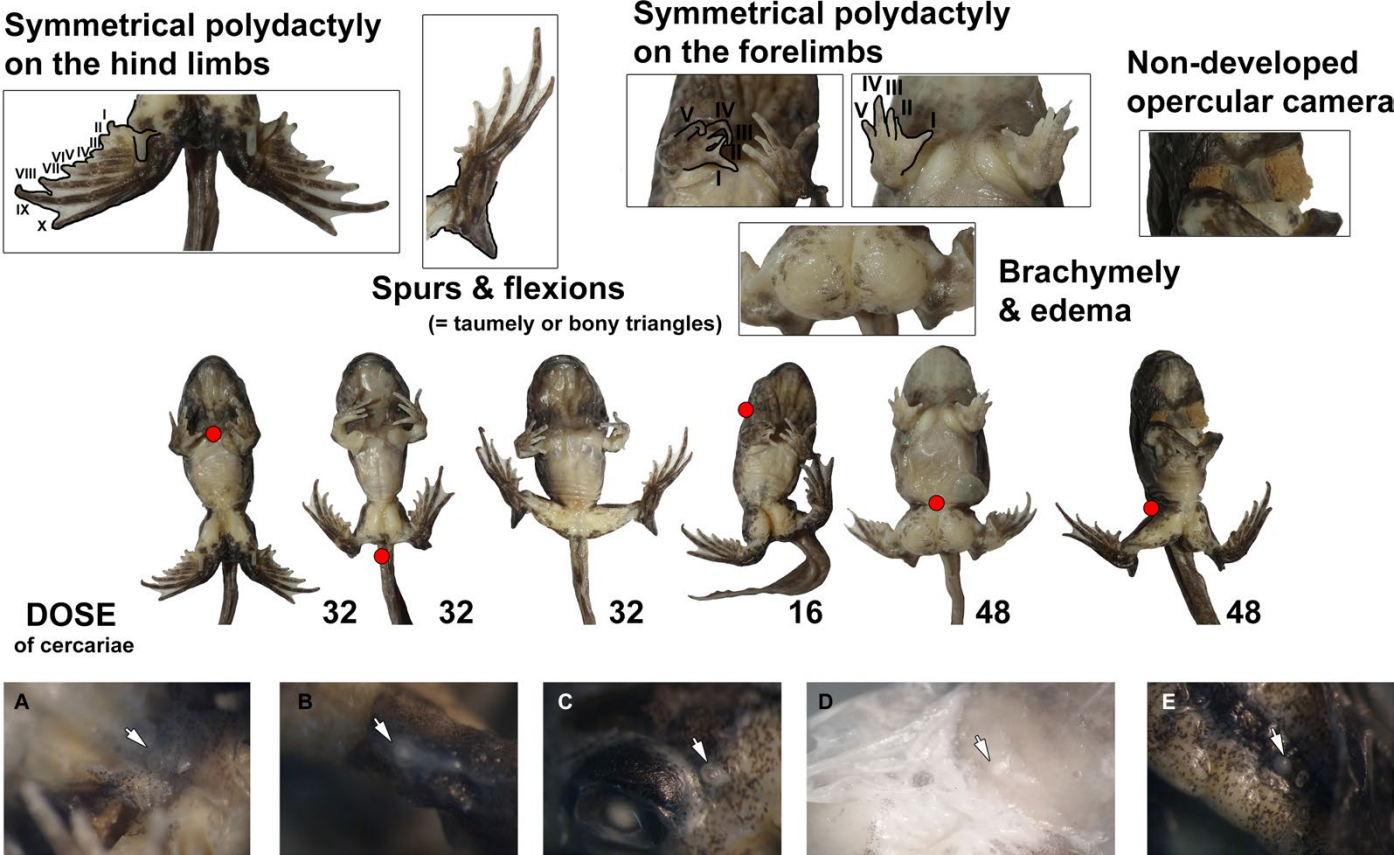
Severe cases of anomaly P in *Pelophylax lessonae* tadpoles under exposure to different doses of *Strigea robusta* cercariae and localization of cysts in tadpoles (arrows)

The tadpoles from the laboratory experiments showed both the severe and mild forms of anomaly P (Fig. 2): 23 (61%) tadpoles had the mild form (polydactyly) of the syndrome, whereas 8 tadpoles (21%) had severe forms of anomaly P. Seven tadpoles (18%) were normal. We registered statistically significant differences in manifestation of anomaly P between groups of treatment for both early (logistic regression with Firth’s correction: *χ*^2^ = 25.08, *df* = 1, Wald = 6.43, *P* = 0.011) and late (*χ*^2^ = 19.11, *df* = 1, Wald = 8.67, *P* = 0.003) groups of tadpoles.

Because we grouped tadpoles of the same treatment together in aquaria after infection, we should check our results in the following way. If stage is ignored for a moment, then there are two replicate aquaria for each exposure dosage, and tadpoles can be statistically nested within aquaria using a random intercept term for the container. We tested the effects of *S. robusta* on survival and anomaly manifestation in a mixed model, where individual tadpoles are used as nested observations. We found a significant negative effect of dose (estimate coefficient = -0.09, *P* < 0.001), and a positive effect of time of exposure (estimate coefficient = 1.43, *P* = 0.027) for survival, while effect of dose was positive (estimate coefficient = 0.15, *P* < 0.001) and insignificant in the case of time of exposure (estimate coefficient = -0.68; *P* = 0.383) for abnormality rates.

The mild form included symmetrical cases of polydactyly with 1 (57%) and 2 (39%) additional digits on the hind limbs. One individual (4%) had asymmetrical polydactyly (7 digits on the right side and 6 on the left side). All tadpoles with 6 digits on the hind limbs (except one) had 4 digits on the forelimbs. Seven tadpoles, which had 7 digits on the hind limbs, also had 5 digits on the forelimbs; one individual with 7 toes had asymmetry on the forelimbs (5 digits on the right side and 4 on the left side) and three 7-digit individuals had 4 digits on the forelimbs. Despite exceptions, we found a strong correlation between the number of digits on the hind- and forelimbs in the mild form of anomaly P (*r* = 0.65, *P* = 0.001).

The severe form included brachymely (shortened femur and tibia), polydactyly, flexions (taumely or bony triangles), and spurs. Two individuals had 6 digits on the hind limbs and 4 digits on the forelimbs, 3 tadpoles had 7 digits on the hind limbs and 4 digits on the forelimbs (but one had an asymmetrical case with five on the right side), and one for each variant with 5 digits on the forelimbs and 8, 9 and 10 (10 on the right side and 9 on the left) digits on the hind limbs. We also found a correlation between the numbers of digits on the fore- and hindlimbs for severe anomalies (*r* = 0.79, *P* = 0.020).

The percentage of individuals with 5 fingers on the forelimbs increased as the dose of cercariae increased: with 16 cercariae there were 16% of such individuals, with 32 cercariae 20% and with 48 cercariae 78%.

It is important to note that 4 cases of asymmetrical polydactyly (2 on the hindlimbs and 2 on the forelimbs; 2 for severe cases and 2 for the mild form) had supernumerary digits always on the right side.

The severe forms of anomaly P developed only in the “early” stages under all doses of cercariae exposure. Polydactyly predominantly developed in the “late” stages; however, under the light dose of exposure, polydactyly also developed in “early” tadpoles. Normal tadpoles were found in the “late” stages under the “light” and “intermediate” doses of exposure only.

*Strigea robusta* cysts were observed under the skin in the tails and limbs of abnormal tadpoles, indicating their successful infection and development into metacercariae. We found 41 cysts in tadpoles before metamorphosis: 14 in tadpoles with severe cases of anomaly P; 26 in the mild form and one cyst in normal individuals (which had received a dose of 16 cercariae); 20–30% of cysts were localized under the skin in the tail, dorsal part of body and head. Some cysts were found on the ventral side in the pectoral and pelvic girdles (Fig. 2). Some polydactyly individuals had a higher number of cysts than some individuals with severe cases of anomaly P, which indicates that the complexity of deformities depends more on localization of cysts than on their number.

Tadpoles reared together with infected snails had different levels of anomaly complexity connected with the period of coexistence. A 3-day period led to polydactyly in 90% of cases. After 20 days of coexistence 5 tadpoles had severe cases of anomaly P and 1 tadpole had polydactyly on the hindlimbs. After 20–30 days of coexistence with infected snails one surviving tadpole had a severe case of anomaly P.

### Field observations

In 2019, we found anomaly P in natural populations (4 cases out of 48 metamorphs and tadpoles, i.e. 8.3%), which indicated that the “infectious agent” was present in natural populations during our laboratory experiments (Rostand noted the disappearance of the anomaly in natural populations [9]). In a total four-year period of research, the anomaly P observed in 22.9% of water frogs (*n* = 432) from field sites (Ostrovtsovskaya Lesosteppe): 18.3% had polydactyly and 4.6% had severe forms of the anomaly. Adult and subadult frogs had 9.8% of the mild form of anomaly P, juveniles and tadpoles had 21.2% of polydactyly and 6.3% of severe forms. We found differences between frequency of anomalies in tadpoles/juveniles and adult/subadult frogs (*χ*^2^ = 4.6, *P* = 0.032); the rate of the anomaly in adult/subadult frogs was lower than in earlier age groups.

The frequency of *P. corneus* infected with *S. robusta* from the same ponds with high a frequency of anomalies was 0.38% (*n* = 1316). *Planorbis planorbis* was infected with *S. robusta* (prevalence of 0.24%; *n* = 851). The differences between the prevalence of *S. robusta* in these two snail species was not significant (*χ*^2^ = 0.04, *df* = 1, *P* = 0.848).

## Discussion

Anomaly P has interested scientists for over 70 years. Since its discovery by Jean Rostand in 1949‒1952, it is known as a syndrome of rare but very peculiar morphological anomalies with unknown etiology occurring in water frogs of the genus *Pelophylax*. Undoubtedly, during this time, “light” forms of anomaly P were observed by biologists, who regarded them as cases of symmetrical polydactyly [10]. However, 50 years after Jean Rostand’s works, this anomaly has never been studied in detail in any laboratory in Europe.

Mass polydactyly in marsh frogs was discovered in Ostrovtsovskaya Lesosteppe water bodies in June 2016 [11]. In August 2016, we found severe forms of anomaly P. The discovery of a high frequency of the anomaly in ponds without fish, which were considered by Rostand as the teratogenic vector of the syndrome, prompted us to study molluscs and test a possible trematode infection hypothesis. During winter 2017‒2018, we obtained anomaly P in the laboratory from tadpoles raised together with the mollusc *P. corneus* [12]. The obtained results encouraged us to consider a species of trematode as a possible infectious agent of anomaly P. Thus, we designed a study on how trematodes from planorbid snails can affect tadpoles in experimental conditions.

**Fig. 3:**
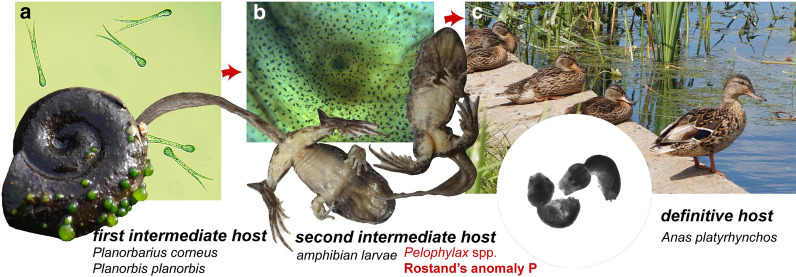
Life-cycle of *Strigea robusta*. **a** First intermediate hosts are planorbid snails (*Planorbarius corneus* and *Planorbis planorbis*). **b** Second intermediate hosts are amphibian larvae and adult amphibians; anomaly P is developed in water frog tadpoles at early stages of development (pre-limb and limb bud stages). **c** Definitive hosts are anatid birds (*Anas platyrhynchos*)

The positive results of experiments with direct cercariae exposure provide compelling evidence that *S. robusta* led to anomaly P in tadpoles of water frogs. The life-cycle of *S. robusta* was investigated in detail by Odening [32] and completed by Vojtek [41]. It has a three-host life-cycle, which includes planorbid snails as first intermediate hosts, larvae of frogs, toads, and newts as second intermediate hosts, and birds of the family Anatidae as definitive hosts (Fig. 3).

As a rule, the first intermediate hosts are freshwater snails of the genera *Planorbis* and *Anisus*. It was experimentally shown that *Bathyomphalus contortus*, *Anisus leucostomus*, *Gyraulus albus*, *Segmentina nitida* and *P. corneus* could also play the role of first intermediate hosts [32, 41]. A total of 1989 *P. corneus* investigated in the Czech Republic [33] as well as 1999 in Belarus [42] did not contain larval stages of *S. robusta*, whereas this species occurred in *Planorbis* and *Segmentina nitida*. Thus, a unique situation was discovered here, in which *S. robusta* was parasitizing *P. corneus* in natural populations.

The second intermediate hosts are larvae of *Lissotriton vulgaris* and *Triturus cristatus* according to Vojtek [41] and Sinsch et al. [40, 43]. Odening [32] reported brown frogs infected with *Strigea robusta* near Berlin, with a prevalence of 60–100%. He conducted experiments with the anurans *Rana arvalis*, *Rana temporaria* and *Bufo bufo*, and obtained metacercariae from these species [32]. No information was provided by this author regarding anomalies in tadpoles of brown frogs or common toads from these experiments. Similarly, there is no information from Vojtek’s [41] experiments about any anomalies in the development of green frog tadpoles. According to our data, the anomaly P developed in four species of water frog; it was found in nature in the marsh frog *P. ridibundus* [11], the Sahara frog, *P. saharicus* [9], and obtained in laboratory hybridogenous *P. esculentus* [12], and in the pool frog *P. lessonae* [4].

In Europe, ducks are the definitive hosts of *S. robusta*. This species was reported to occur in 3% of *Anas platyrhynchos* and 0.9% of *Aythya fuligula* [44]. According to Sudarikov [45], a wider spectrum of birds can be infected with *S. robusta*.

Rostand reported the existence of a sensitive period for the action of the infectious agent during the early stages of tadpole development [9]. We found that anomalies developed in tadpoles up to Gosner stage 36; after that, anomalies did not develop in tadpoles. The complexity of the anomalies (light or severe forms) depended on the stage of the tadpole: tadpoles at “late” 29‒34 Gosner stages, developed polydactyly only, whereas tadpoles at “early” 25‒27 Gosner stages, developed severe forms of anomaly P. According to our data, the complexity and severity of the anomalies may also depend on the number of metacercariae, their localization, and individual immunological sensitivity. Because we detected cysts after preservation of tadpoles in 70% of ethanol, data about the number of metacercariae that survived is incomplete and further investigation of this question is required.

The limbs of other amphibians that live syntopically and are infected with *S. robusta* show no anomalies. In Russia, these are *Lissotriton vulgaris*, *Triturus cristatus*, *Pelobates vespertinus*, *Bombina bombina* and *Rana arvalis* [11]. Possibly, it can be explained by differences in terms of reproduction and development of tadpoles (especially, development of their limb buds) from one side, and the time of sporocysts development and cercariae emergence, from another. However, *B. bombina* has a period of reproduction and duration of larval development similar to *P. ridibundus*. It is very interesting to note that in introduced populations of *P. ridibundus* in the Ural Region polydactyly has also been detected [46] and can be caused by *S. robusta*. Probably, *S. robusta* may have been introduced together with *P. ridibundus*, but more likely it has switched to a new host, because this species was registered east of the Ural Mountains. For example, *S. robusta* was found in Siberia [45, 47], and in a Siberian population of the moor frog, *R. arvalis*, cysts of *S. robusta* were found in 2.5% of studied frogs [47]. Obviously, *S. robusta* can live outside of water frog ranges and have a life-cycle not involving *Pelophylax* spp. Literature data indicate that the main second intermediate hosts for this species are brown frogs of the genus *Rana* [32, 45]. Possibly, *S. robusta* can switch between amphibian hosts, but its teratogenic effects for other species of amphibians remain unknown, and such long-term co-evolutionary relationships of *S. robusta* with different amphibian hosts are of great interest. Also, we were not aware of any records of anomalies similar to severe cases of anomaly P in other amphibian species.

Therefore, the teratogenic effects appear to be taxon specific. The teratogenic factor was supposed to be retinoic acid in the trematode *Ribeiroia ondatrae* [48]. In the case of anomaly P, the teratogenic factor can influence the expression levels of *HoxD* genes [49, 50] and lead to a peculiar morphology with the reduction of the hind limb’s proximal parts (brachymely) and multiplication of the distal parts (polydactyly). Some similar anomalies in vertebrates caused by deletions in *PITX1* and represented by a spectrum of lower-limb malformations including mirror-image polydactyly [51].

What effect can this trematode have on amphibian populations? The invasion of *S. robusta* led to the decline of a large smooth newt population (*Lissotriton vulgaris*) inhabiting a pond at Schmidtenhöhe near Koblenz [43]. It is important to note, that none of the crested newt (*Triturus cristatus*) populations, was infected with *S. robusta*, whereas 73% of the smooth newts were infected [40, 43]. Differences in infections with metacercariae in adult *Lissotriton* newts and larvae (that had no cysts), in various species of newts (*T. cristatus* and *L. vulgaris*), as well as infection rates between populations of brown frogs, are still unknown and intriguing [43]. Finally, it has been proven that *S. robusta* reduced the survival of newts and led to an exceptional case of population decline in amphibians under trematode infection [40, 43].

Our data confirm this information, showing that under the influence of *S. robusta* the survival rates of water frog tadpoles decrease, and that infection with metacercariae leads to the manifestation of a polymorphic syndrome, the extreme manifestation of which is grotesque deformations incompatible with life. The rate of anomaly P in water frog populations can reach 80% (severe cases can reach 40–50% in abnormal cases) according to Rostand’s data [8–11], 23% according to our field data and 82% according to data obtained in the laboratory infection of tadpoles. From an ecological point of view, it may be interesting to understand whether a decrease in locomotor activity and survival of a large number of tadpoles may lead to the decline and extinction of water frog local populations. Probably, the balance between normal forms and abnormal specimens in water frog populations may result from the limited time span in the early stages of tadpole development and from the rather rare occurrence of *S. robusta* in planorbid molluscs.

Thus, many questions remain unanswered. The possibility of species of the genus *Strigea* to cause anomalies in amphibians is of great interest. Currently, more than 50 species of *Strigea* are known in Africa, Australia, Europe, Asia, and North and South America [52, 53], and the phylogenetic relationships within this group remain unknown. *Strigea robusta* may prove of considerable interest for the study of molecular and physiological mechanisms of trematode action on their intermediate hosts.

## Conclusions

Cercariae dose-dependent experiment clearly indicates that Rostand’s mysterious anomaly P syndrome in water frogs is caused by the trematode *S. robusta*. The complexity of anomalies depends on stages of limb development in tadpoles. At the early stages, severe or mild forms of anomaly P developed, while at the late stages we observed polydactyly only. We also confirmed the rare occurrence of *S. robusta* in planorbid snails in wildlife and found a unique situation, in which *S. robusta* was parasitizing *P. corneus*. Anomaly P syndrome is a new variant of morphological anomalies in amphibians caused by a trematode species. Nowadays, mass polydactyly cases in western Palaearctic water frogs observed over Europe can be partially explained by the action of this species.

## Data Availability

Our sequences were deposited in the GenBank database under the accession numbers MT075803, MT075804, MK295776, MK295777 (ITS2) and MT075841, MT075842, MK585229, MK585230 (*28S* rRNA).

## Abbreviations

PCR: polymerase chain reaction
dNTP: deoxyribonucleotide triphosphate
ITS2: internal transcribed spacer 2
*PITX*1: pituitary homeobox 1
*HoxD*: homeobox D cluster
